# Pulmonary Tuberculosis Presenting as Severe Acute Respiratory Distress Syndrome in a Capsule Hotel Resident

**DOI:** 10.7759/cureus.80082

**Published:** 2025-03-05

**Authors:** Ichita Yamamoto, Tomoya Okazaki, Yasuhiro Norisue

**Affiliations:** 1 Department of Emergency and Critical Care Medicine, Tokyo Bay Urayasu Ichikawa Medical Center, Urayasu, JPN

**Keywords:** active pulmonary tuberculosis, acute respiratory distress syndrome (ards), clinical case report, extracorporeal membrane oxygenation support, medical intensive care unit (micu), pulmonary and critical care medicine, severe pneumonia, severe respiratory failure, tuberculosis differential diagnosis, vv-ecmo

## Abstract

We report a case of a 60-year-old man who developed severe respiratory failure after collapsing in a sauna facility. Initially diagnosed with severe community-acquired pneumonia and acute respiratory distress syndrome (ARDS), his condition deteriorated despite standard treatment, necessitating venovenous extracorporeal membrane oxygenation (VV-ECMO). Acid-fast bacilli testing confirmed pulmonary tuberculosis (TB) on day 4, prompting the initiation of anti-TB therapy. Following clinical improvement, the patient was successfully weaned from VV-ECMO and transferred to a specialized TB hospital for ongoing care. This case highlights the importance of considering TB in cases of antibiotic-refractory severe pneumonia and ARDS, particularly in patients with social risk factors, even in low-incidence countries. Early recognition and treatment of TB are crucial to improving patient outcomes and minimizing transmission risks.

## Introduction

Pulmonary tuberculosis (TB) often presents with subacute or chronic symptoms but can occasionally cause acute respiratory distress syndrome (ARDS), necessitating intensive care [1-3]. Although TB incidence in Japan is low (<10 cases/100,000/year), it persists as a significant public health concern, particularly in overcrowded or poorly ventilated environments [4-6].

This report describes a patient with TB-induced ARDS initially misdiagnosed as severe community-acquired pneumonia and lung abscess. It highlights the importance of considering TB in cases of severe respiratory failure unresponsive to antibiotics, even in low-incidence settings.

## Case presentation

A 60-year-old man collapsed in the sauna facility of a capsule hotel, a budget accommodation facility with small sleeping units, where he had resided for the past two years. The patient reported a one-week history of poor appetite, diarrhea, and low-grade fever, accompanied by progressively worsening dyspnea over the three days prior to admission. His medical history and medications were unknown due to a lack of family contacts and severe dyspnea that prevented a detailed history taking.

On admission, the patient presented with tachycardia, tachypnea, and hypoxemia. His vital signs were as follows: blood pressure 128/85 mmHg, heart rate 120 beats per minute, respiratory rate 40 breaths per minute, oxygen saturation 88% on a 15L non-rebreather mask, and body temperature 37.8°C, consistent with low-grade fever. Physical examination revealed rapid, shallow breathing using accessory respiratory muscles. His Glasgow Coma Scale score was E4V5M6.

**Figure 1:**
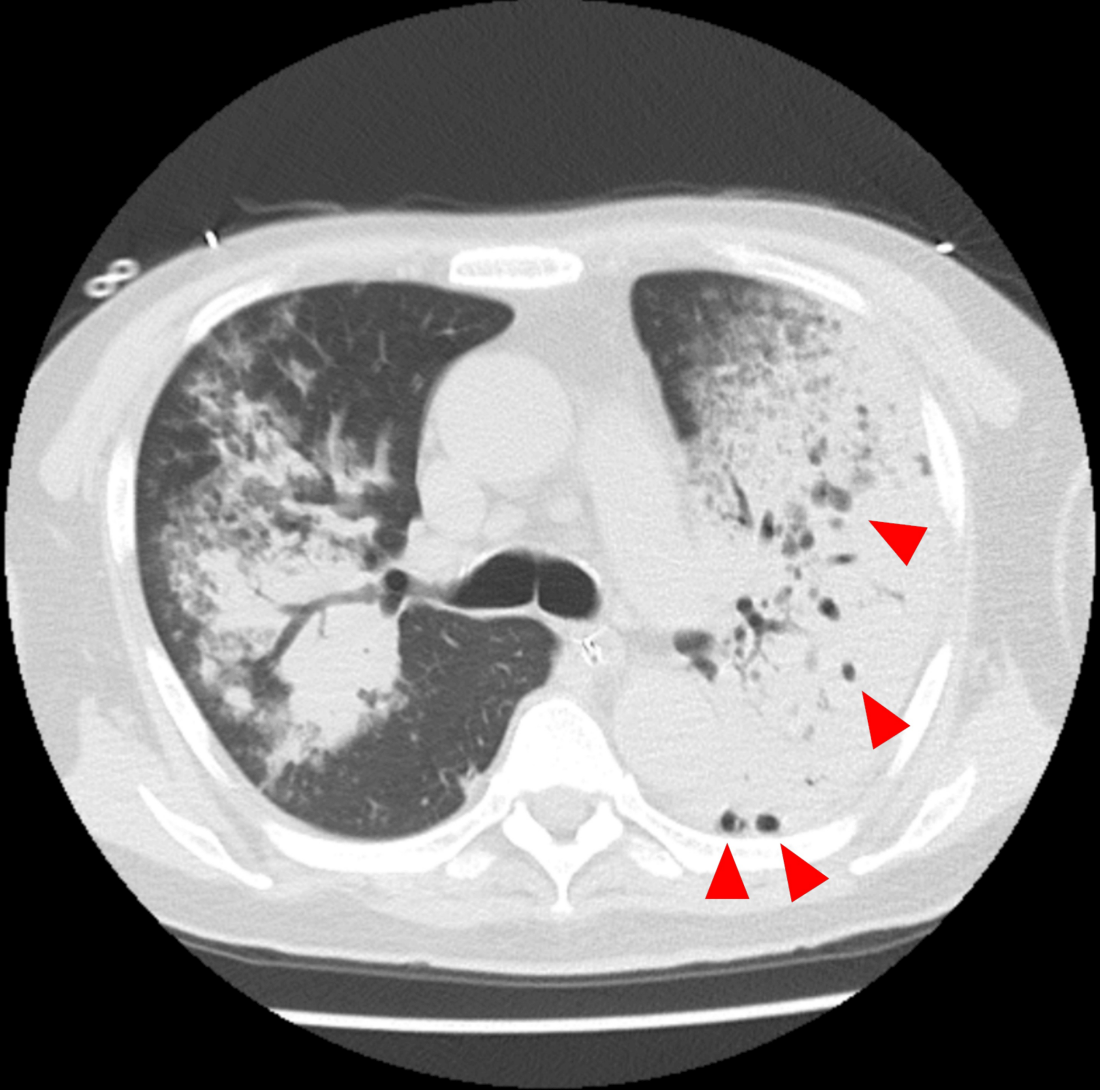
Contrast-enhanced chest CT showing bilateral consolidations and cavitary lesions (red arrowheads) predominantly in the left lung CT: computed tomography

Initial laboratory investigations revealed severe metabolic acidosis with respiratory compensation, as evidenced by an arterial blood gas analysis showing a pH of 7.067, partial pressure of carbon dioxide (pCO₂) of 63.1 mmHg, partial pressure of oxygen (pO₂) of 126 mmHg, bicarbonate (HCO₃⁻) of 17.3 mmol/L, and lactate of 11 mmol/L. Laboratory results also indicated severe renal dysfunction with a blood urea nitrogen (BUN) of 147.2 mg/dL and creatinine of 8.82 mg/dL. Aspartate aminotransferase (AST) was elevated to 268 U/L and alanine aminotransferase (ALT) to 108 U/L, along with lactate dehydrogenase (LDH) at 1910 U/L and creatine kinase (CK) at 1552 U/L. Marked inflammation was evident, with a white blood cell (WBC) count of 64 × 10⁹/L and a C-reactive protein (CRP) level of 17.13 mg/dL. The patient also had thrombocytopenia, with a platelet (PLT) count of 8.6 × 10⁹/L (see Table 1 for full laboratory results). Tests for HIV, COVID-19, and urine Legionella antigen were negative. A chest CT scan revealed bilateral consolidations with cavitary lesions, predominantly in the left lung (Figure 1). 

**Table 1:** Blood test results eGFR: estimated glomerular filtration rate, CRP: C-reactive protein, BNP: brain natriuretic peptide

Laboratory tests	Value	Unit	Reference ranges
White blood cell	64	× 10^9/L	40-80
Hemoglobin	14.9	g/dL	14.0-18.0
Platelets	8.6	× 10^9/L	15.0-35.0
Blood urea nitrogen	147.2	mg/dL	8.0-22.0
Creatinine	8.82	mg/dL	0.61-1.04
eGFR	4	mL/min	60-
Aspartate aminotransferase	268	U/L	13-33
Alanine transaminase	108	U/L	8-42
Alkaline phosphatase	116	U/L	38-113
Lactate dehydrogenase	1910	U/L	119-229
Creatine kinase	1552	U/L	60-287
Total bilirubin	1.38	mg/dL	0.2-1.2
Sodium	130	mmol/L	138-146
Potassium	4	mmol/L	3.6-4.9
Chloride	98	mmol/L	99-109
Calcium	6.6	mg/dL	8.7-10.3
Magnesium	3.9	mg/dL	1.9-2.5
Phosphate	10.2	mg/dL	2.5-4.7
CRP	17.13	mg/dL	0-0.29
BNP	24.7	pg/mL	0-18.4

Sputum samples obtained via bronchoalveolar lavage revealed both Gram-positive cocci and Gram-negative rods. The patient was diagnosed with severe pneumonia and lung abscess caused by a polymicrobial infection. He was intubated upon ICU admission. Empiric antibiotics, including vancomycin, piperacillin-tazobactam, and azithromycin, were initiated. Hydrocortisone 200 mg/day was also administered. The initial PaO₂/FiO₂ ratio was 110 on mechanical ventilation, consistent with moderate ARDS. Due to worsening hypoxia, neuromuscular blockade was started and continued for 48 hours, and prone positioning was initiated. Due to worsening renal dysfunction, renal replacement therapy was initiated. A permissive hypercapnia strategy allowed a pH as low as 7.15.

By the third day, his PaO₂/FiO₂ ratio had rapidly declined to 65, showing further deterioration. Inhaled nitric oxide therapy was initiated but did not result in significant improvement. Consequently, venovenous extracorporeal membrane oxygenation (VV-ECMO) was initiated on the evening of day three.

Sputum cultures yielded only *Moraxella catarrhalis*, which was discordant with the initial Gram stain, suggesting polymicrobial infection. The culture results were inconsistent with the initial Gram stain findings. Considering this discrepancy and the patient's residence in a capsule hotel, TB was suspected as the underlying cause of his refractory pneumonia and ARDS. On day four of admission, acid-fast bacilli testing was strongly positive (>10 acid-fast bacilli per oil immersion field), and polymerase chain reaction detected *Mycobacterium tuberculosis*, confirming the diagnosis of pulmonary TB. Anti-TB therapy with isoniazid, rifampicin, pyrazinamide, and ethambutol was initiated. Methylprednisolone 1 g was administered for three days to address TB-associated ARDS. A daily bronchoscopy was performed to manage sputum clearance.

**Figure 2:**
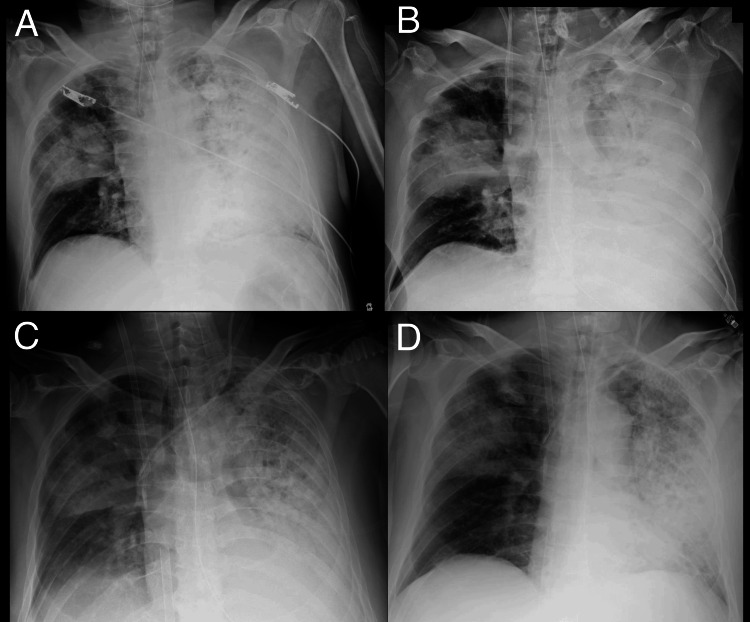
Serial chest radiographs showing the progression and recovery of ARDS in TB A chest X-ray taken upon emergency department arrival, immediately after intubation, showed reduced transparency in the right lung and diffuse opacities in the left lung (A). On the night of day 3, prior to the initiation of VV-ECMO, progressive consolidation was noted in the left lung (B). On day 9, bilateral lung opacities persist (C). On day 11, at the time of VV-ECMO removal, marked improvement in bilateral lung opacities is evident (D). ARDS: acute respiratory distress syndrome, TB: tuberculosis, VV-ECMO: venovenous extracorporeal membrane oxygenation

**Figure 3:**
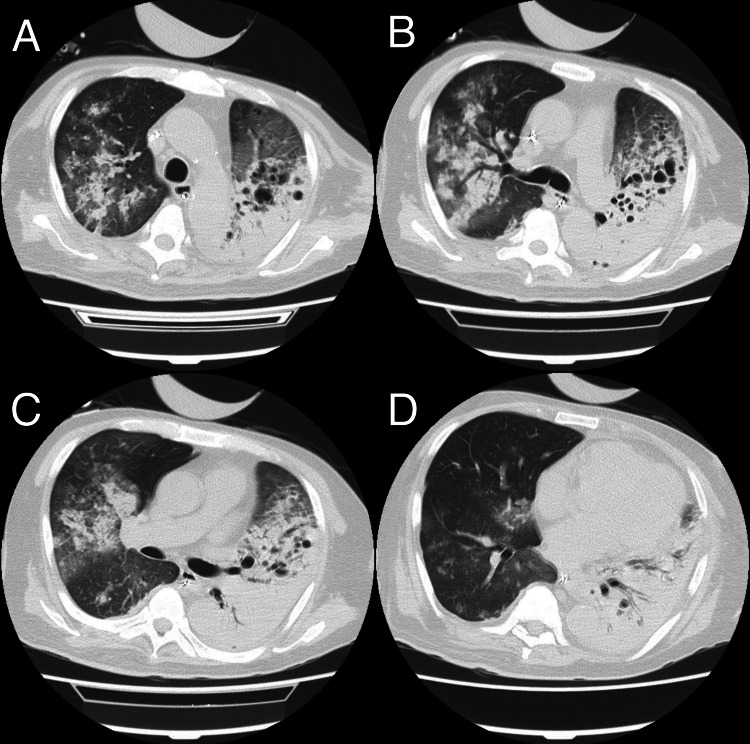
Chest CT on day 13 showing resolution of pulmonary infiltrates in ARDS in TB Chest CT images on day 13 demonstrate partial resolution of consolidations, though bilateral consolidations and multiple cavitary lesions in the left lung are still present (A, B, C, and D). CT: computed tomography, ARDS: acute respiratory distress syndrome, TB: tuberculosis

Between days 9 and 11, the patient’s clinical condition improved, as evidenced by radiographic resolution of pulmonary infiltrates (Figure 2 and Figure 3) and an increase in respiratory system compliance to 40 mL/cmH₂O, enabling successful weaning from VV-ECMO. Subsequently, a tracheostomy was performed five days later to facilitate prolonged ventilatory support.

**Figure 4:**
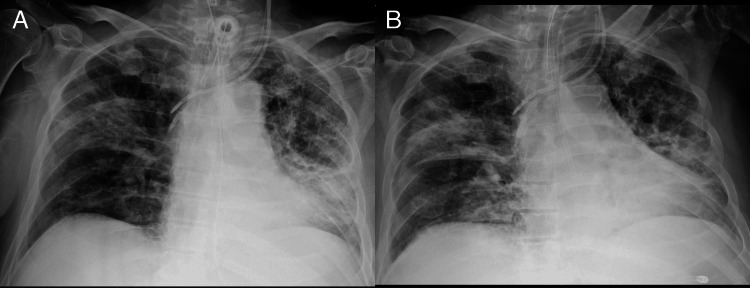
Serial chest radiographs showing late-stage recovery after VV-ECMO decannulation in ARDS due to TB A chest X-ray taken on day 21, 10 days after VV-ECMO decannulation, showed further resolution of bilateral lung opacities and ongoing lung recovery (A). On day 28, just before discharge, imaging revealed little further resolution, with residual lung opacities still present (B). VV-ECMO: venovenous extracorporeal membrane oxygenation, ARDS: acute respiratory distress syndrome, TB: tuberculosis

Two days after the tracheostomy, drug-induced liver injury necessitated adjustment of anti-TB therapy to ethambutol, streptomycin, and levofloxacin. Cycloserine was added three days later. The patient remained ventilator-dependent and was transferred to a designated TB hospital 29 days after admission (Figure 4). Since multiple healthcare workers were exposed to TB before its confirmation, an exposure screening program was implemented. This included medical evaluation, chest radiography, and interferon-gamma release assay testing without identifying active or latent TB infections among the staff.

## Discussion

Pulmonary TB usually presents with sub-acute or chronic symptoms. However, TB can present acutely or rapidly with severe respiratory failure and ARDS, making the initial diagnosis challenging. In our case, despite the initial diagnosis of severe community-acquired pneumonia with lung abscess and implementation of standard antibiotic therapy with standard treatment for ARDS, the patient's condition progressively deteriorated to require VV-ECMO support. This case highlights the importance of considering TB in the differential diagnosis of severe pneumonia, even when initial radiological findings suggest bacterial pneumonia. Although TB was not initially suspected due to the patient’s acute presentation and polymicrobial findings on Gram stain, it was later considered in light of his prolonged residence in a high-density communal living environment. Earlier suspicion and targeted testing in high-risk individuals, particularly when initial treatment for bacterial pneumonia proves ineffective, may facilitate timely diagnosis and reduce the risk of transmission.

The epidemiology and outcomes of severe TB requiring ICU admission vary across studies. Galvin et al. reported in their systematic review that respiratory failure and ARDS were the most common indications for ICU admission (36.3%), with ARDS affecting 19.5% of all cases [1]. They also reported delays in diagnosis and highlighted the challenge of differentiating TB from severe bacterial pneumonia as a cause of ARDS. The mortality of these severe cases is notably high, with Muthu et al.'s systematic review reporting a pooled ICU mortality of 48% and hospital mortality of 54% in patients with TB requiring intensive care [1,2]. Early TB recognition in critically ill patients presents a significant diagnostic challenge, complicating clinical management. Valade et al.'s single-center study in France found that among 53 ICU patients over a 10-year period, 13 were diagnosed with TB only after ICU discharge, with eight of these initially misdiagnosed as bacterial pneumonia. The authors emphasize the importance of considering TB in cases of severe pneumonia, particularly when patients have a history of prolonged respiratory symptoms before ICU admission [6]. In our case, the patient had a brief symptom presentation and an unknown medical background. Gram stain and culture also showed pathogens consistent with community-acquired pneumonia, making the initial diagnosis challenging.

A recent 2024 international multicenter study systematically analyzed 79 cases of TB-induced ARDS treated with ECMO over a 20-year period [7]. This study reported an overall 90-day survival rate of 51%, with several factors associated with poor outcomes: advanced age, drug-resistant TB, and higher pre-ECMO sequential organ failure assessment (SOFA) scores. Our patient presented with significant organ dysfunction (SOFA score of 11), including severe renal failure requiring continuous hemodiafiltration and marked thrombocytopenia, and was relatively elderly (60 years old). However, the relatively early initiation of anti-TB therapy may have contributed to the initial successful weaning from ECMO support. The duration of ECMO in our case (11 days) was shorter than the median duration reported in the multicenter study (20 days), likely due to the relatively early initiation of anti-TB therapy following microbiological confirmation, which led to rapid clinical improvement.

This case demonstrates the importance of considering TB in the differential diagnosis of ARDS, even in a low-incidence country like Japan. Although Japan has been classified as a low-incidence country for TB since 2021 [5], TB remains a significant public health concern, particularly among vulnerable populations exposed to high-risk environments [4]. The recent international multicenter study also highlighted the significance of social factors, reporting that among TB patients requiring ECMO support, 27% were migrants, and 5% were from unstable housing conditions, including homelessness [7]. These findings emphasize the necessity of considering TB early in individuals from high-risk environments, even when their initial presentation mimics bacterial pneumonia.

Capsule hotels, a budget accommodation facility popular among business travelers and tourists, feature small sleeping units with shared bathrooms and common areas. While primarily designed for short-term stays, these facilities have also become long-term residences for some individuals. The communal nature of these facilities, combined with limited ventilation in individual units and shared spaces like saunas, creates an environment conducive to TB transmission. While TB spreading among individuals experiencing homelessness is a recognized issue in Japan, there are also individuals who, though not living on the streets, lack conventional housing and stay in capsule hotels or internet cafes [8,9]. Some individuals experience unstable housing conditions and rely on day labor for income. When they earn daily wages, they may stay in capsule hotels or internet cafes; however, when they do not, they are often left without stable shelter and may sleep on the streets.

Furthermore, the Ministry of Health, Labour and Welfare of Japan has reported TB cluster infections in capsule hotels, saunas, and simple lodging facilities [10]. However, specific epidemiological data regarding TB prevalence among capsule hotel residents remains unavailable. Given the recent increase in inbound travelers and foreign workers in Japan, some of whom may come from TB-endemic regions, further public health investigations are warranted to better understand TB transmission risks in these environments. Raising awareness among healthcare providers about the potential for TB in this demographic may facilitate earlier diagnosis and reduce transmission risks.

## Conclusions

In cases of severe respiratory failure unresponsive to standard antibiotics, TB should be considered in the differential diagnosis, particularly in individuals from shared or enclosed living environments. Even in low-incidence countries, failing to recognize TB early may lead to delayed diagnosis, worsening clinical outcomes, and increasing the risk of transmission, including to healthcare workers. Awareness of social and epidemiological risk factors is essential to prompt recognition and timely intervention, which are key to improving patient care and preventing nosocomial spread.
